# Challenges in Surgical Training in the United Kingdom and Potential Solutions: A Narrative Review

**DOI:** 10.7759/cureus.106815

**Published:** 2026-04-10

**Authors:** Hammaad Khalid, Josephine Walshaw, Marina Yiasemidou

**Affiliations:** 1 Medicine, Leeds Teaching Hospitals NHS Trust, Leeds, GBR; 2 Surgery, Hull University Teaching Hospital, Hull, GBR; 3 Colorectal Surgery, The Royal London Hospital, Barts Health NHS Trust, London, GBR

**Keywords:** medical education, mentorship, nhs, policy, simulation training, surgical training, surgical workforce, uk, united kingdom

## Abstract

Surgical training in the United Kingdom (UK) has undergone a significant transformation in response to changing healthcare needs, reforms, and advancements in medical education. Traditionally centred on an apprenticeship model of supervised clinical learning, modern surgical training faces several challenges that threaten its sustainability. Reduced working hours following the introduction of the European Working Time Directive (EWTD), increasing subspecialisation, and growing service provision pressures within the National Health Service (NHS) have limited operative exposure and training opportunities. Rising competition for surgical training posts has also created a bottleneck within the training pathway, contributing to stress and burnout amongst aspiring surgeons. Additionally, concerns also remain regarding financial barriers and limited diversity within the surgical workforce. This study was conducted as a narrative review of the literature using PubMed to identify studies and policy reports examining challenges in UK surgical training. Potential strategies identified include expanding training capacity, strengthening mentorship and support systems, improving workforce planning, and incorporating simulation and other technological innovations to enhance surgical education. Addressing these issues is essential to ensure a sustainable and well-trained surgical workforce capable of meeting future healthcare demands.

## Introduction and background

In recent decades, surgical training has experienced transformational change in both educational methodologies and technological innovation. Traditionally, the apprenticeship model has formed the cornerstone of surgical education, whereby trainees learn through a "see one, do one, teach one" approach under the mentorship of experienced professionals [1]. This method involves observing surgical procedures and then performing them with guidance from skilled practitioners. It has played a vital role in the transfer of surgical knowledge and expertise from one generation of surgeons to the next [2,3].

However, with rapid progress in medical education and technology, new approaches have emerged to complement and enhance the traditional apprenticeship model [4]. These advancements aim to address some of the challenges associated with relying solely on traditional methods, such as limited access to diverse surgical cases, potential patient risks during trainee-operated procedures, and variability in the quality of training experiences [5]. This narrative review explores the major challenges affecting surgical training in the UK and discusses potential strategies to improve training quality, workforce sustainability, and trainee well-being.

Methods

This article was conducted as a narrative review examining current challenges and potential solutions in surgical training within the UK. A literature search was performed using electronic databases, including PubMed, to identify relevant studies, reports, and policy documents relating to surgical education and workforce planning. Additional sources were identified through screening the reference lists of relevant publications and reports from professional bodies such as the General Medical Council (GMC) and the Royal College of Surgeons (RCS). Key search terms included surgical training, surgical education, United Kingdom, UK, surgical workforce, competition ratios, simulation training, and diversity in surgery. Articles were selected based on their relevance to key themes affecting surgical training, including reduced operative exposure, competition for training posts, workforce pressures, trainee well-being, and diversity within the surgical profession.

Studies were included if relevant to surgical training in the UK and addressed the above themes. Articles not relevant to the UK or not written in English were excluded. Study selection began with screening of titles and abstracts, then full-text review where appropriate. As this was a narrative review, no formal risk-of-bias assessment was done. Evidence was synthesised using a thematic narrative approach to summarise key findings.

## Review

Limitations of the traditional apprenticeship model

Several interrelated structural, educational, and workforce challenges continue to affect the quality, accessibility, and sustainability of surgical training in the UK. In the past, feedback provided by senior surgeons has been indispensable in transferring the cognitive knowledge essential for developing technical surgical skills [2]. This apprenticeship model has traditionally served as the cornerstone of surgical education, fostering a hands-on learning experience under the guidance of experienced mentors.

However, despite its historical significance, the traditional apprenticeship model is no longer fully sufficient due to several factors. One such factor is the proliferation of subspecialties within the field of surgery. With the emergence of more specialised areas, the breadth of surgical knowledge required of trainees has expanded considerably. This makes it increasingly challenging to cover all subspecialties comprehensively via a traditional apprenticeship model. Moreover, service provision has become a recurring issue in the modern healthcare landscape, affecting both senior surgeons and trainees alike [6,7]. The increasing workload may impact the quality and availability of one-to-one mentoring and feedback, which are vital components of the apprenticeship style of training.

The introduction of the European Working Time Directive (EWTD) also reduced the overall training hours. The EWTD is now incorporated into UK law, which regulates the working hours of healthcare professionals, including doctors and nurses, to ensure patient safety and the well-being of medical practitioners. The directive imposes limits on the maximum number of working hours, minimum rest periods, and breaks for healthcare workers. While the EWTD is primarily aimed at improving patient safety by reducing the risk of medical errors caused by fatigue and overwork among healthcare professionals, it can also have the unintended consequence of reducing exposure in the operating theatre by approximately 30% [8]. Table 1 summarises these key challenges.

**Table 1:** Key challenges affecting surgical training in the UK. Summary of major structural and educational challenges affecting surgical training in the UK. This table was independently created using information summarised from the cited sources [6-8].

Challenge	Description	Impact
Reduced operative exposure	Working hour restrictions, such as the European Working Time Directive, limit the time spent in the theatre	Fewer opportunities to develop technical skills
Increasing competition ratios	A growing number of applicants are competing for limited training posts	Bottleneck in training progression and career uncertainty
Service provision pressures	NHS demands limit protected training time	Reduced mentorship and teaching opportunities
Financial barriers	Costs associated with courses, exams, and training requirements	Disadvantage(s) trainees from lower socioeconomic backgrounds
Diversity and inclusivity issues	Underrepresentation of women and individuals from disadvantaged backgrounds	Reduced diversity within the surgical workforce
Trainee burnout	High workload and competitive training environment	Reduced well-being and potential attrition from surgical careers

Increasing competition for training posts

Limited training capacity within the UK, combined with increasing demand for surgical training positions, represents a significant challenge for surgical trainees [9]. This results in high competition ratios and can be particularly frustrating for aspiring surgeons [10]. Surgical training programmes, like many specialised medical training programmes, have a fixed number of positions available. These places are determined by various factors, including funding, availability of experienced faculty, and the capacity of healthcare institutions [10]. As the medical field attracts more aspiring surgeons, the demand for training places has intensified, fostering a highly competitive environment [10]. Finally, surgical training is often longer and more intensive than training in many other medical specialities, which further strains the capacity of training programmes [11].

This heightened competition raises legitimate concerns about the future of the UK surgical workforce, especially considering the anticipated rise in demand for surgical services due to the ageing global population [12]. Meeting this expanding demand is contingent on recruiting an adequate number of highly qualified surgeons capable of delivering safe and effective interventions [6]. Unfortunately, the number of available surgical training positions in the UK has failed to keep pace with the increasing number of UK and international medical graduates seeking training opportunities [11]. Restrictions in National Health Service (NHS) funding pose challenges to the expansion of surgical training posts. The financial shortage of £7 billion in the fiscal year 2023-2024, coupled with increasing demands for healthcare services due to factors like COVID-19, inflation, and staff salary settlements, affects the allocation of resources for aspiring surgeons' training opportunities [13]. These budgetary restrictions create opportunity costs and may limit the capacity to address the growing demand for surgical services.

**Figure 1:**
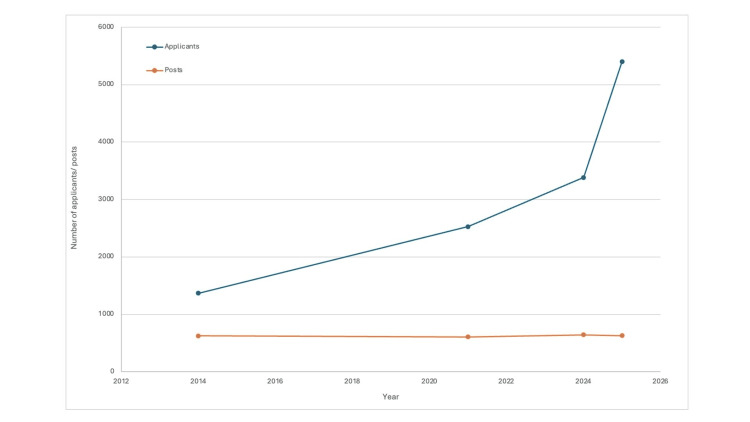
Applicants versus available CST posts in the UK (2014-2025). Trends in Core Surgical Training (CST) applicants versus available training posts in the UK. This figure was created using data extracted and summarised from the cited sources [11,14]. This image was generated using Microsoft Excel (Microsoft Corporation, Redmond, WA, USA).

This disparity between supply and demand has inadvertently created a bottleneck in UK surgical training, leading to an increased number of candidates applying for the same number of surgical posts. This is despite the mounting demand for increased surgical services. Within a span of six years, the competition ratio for Core Surgical Training positions has almost doubled, rising from 2.31 in 2015 to 4.16 in 2021, with 2,528 candidates applying for 607 positions (Figure 1) [14]. The situation is even more challenging for Speciality Training Year 3 (ST3) training positions. In 2011, 2,178 applicants applied for 341 posts, resulting in an overall competition ratio of 6:1 [15]. The repercussions of this bottleneck extend beyond the training process and impact the wider NHS. Striving against a backlog of cases post pandemic, the NHS may encounter hurdles in delivering timely care, resulting in extended waiting lists and potential compromises in patient care [6].

While competition can attract exceptional candidates, it also poses challenges for talented individuals who may feel discouraged or overwhelmed by the rivalry [16]. The high levels of stress and burnout experienced by candidates pursuing surgical specialities are concerning and have significant implications for the healthcare system. In 2020, mean burnout and secondary traumatic stress ratings among surgical candidates in the UK were 38% and 112% higher than population norms, respectively [17]. This statistic is alarming because burnout has been linked to unfavourable effects, including compromised patient safety, causing iatrogenic injuries, and higher rates of professionals leaving the field [16,18]. The presence of stress and burnout also exacerbates staffing challenges, wastes allocated funds, and has adverse effects at both local and national levels [19]. The high levels of stress and burnout may drive some surgical trainees to seek better work-life balance abroad, contributing to “brain drain” and increasing disparities within the UK surgical workforce. This cycle of trainees leaving to optimise working conditions elsewhere may further strain the already challenging environment within the UK, impacting patient care and aggravating the pre-existing challenges [18].

Diversity, inclusivity, and financial barriers

The review has highlighted other issues, including diversity and inclusion, which warrant action within the surgical workforce. Ethical concerns regarding the fairness and transparency of the selection process for surgical training have come to light [20]. The Royal College of Surgeons (RCS) of England states that the ratio of male to female surgical consultants in the UK is approximately 8:1 [21]. Surgical specialities have the lowest proportion of female consultants, and gender parity in surgery is predicted to be reached in decades rather than years. The underrepresentation of women leads to fewer role models for aspiring surgeons [20]. For example, trauma and orthopaedics has a female consultant representation of 7.3%, followed by neurosurgery at 8.2%. Both specialities are expected to reach gender parity by the year 2070 [22]. In contrast, ophthalmology has one of the highest representations of female consultants at 32%. When compared to medical specialities, surgery falls short as 43% of the consultants in medicine are female [23].

The UK Surgical Training application process involves reviewing candidate portfolios, interviews, and situational judgement examinations to select trainees [24]. However, this process may not effectively assess the full range of abilities and qualities required for success in surgical training. For example, relying on specific "tick boxes" to evaluate a candidate's surgical ability may favour individuals with access to supportive networks, thereby introducing biases into the selection process [25].

Enhancing inclusivity in medical school admissions is a potential starting point, as students from underprivileged backgrounds often lack accessible resources and experiences that could strengthen their medical school applications [26]. Throughout medical school, these students may face disadvantages due to their unfamiliarity with the "hidden curriculum", which can create barriers to pursuing surgical careers [27]. Networking plays a crucial role in securing mentorship and guidance for developing a surgical portfolio; however, students who are first in their families to study medicine may not have access to established networks with surgeons, putting them at a further disadvantage [28]. This can lead to feelings of inadequacy and imposter syndrome, which may deter diverse candidates from applying to surgical training, further exacerbating the lack of representation within the field [26].

**Figure 2:**
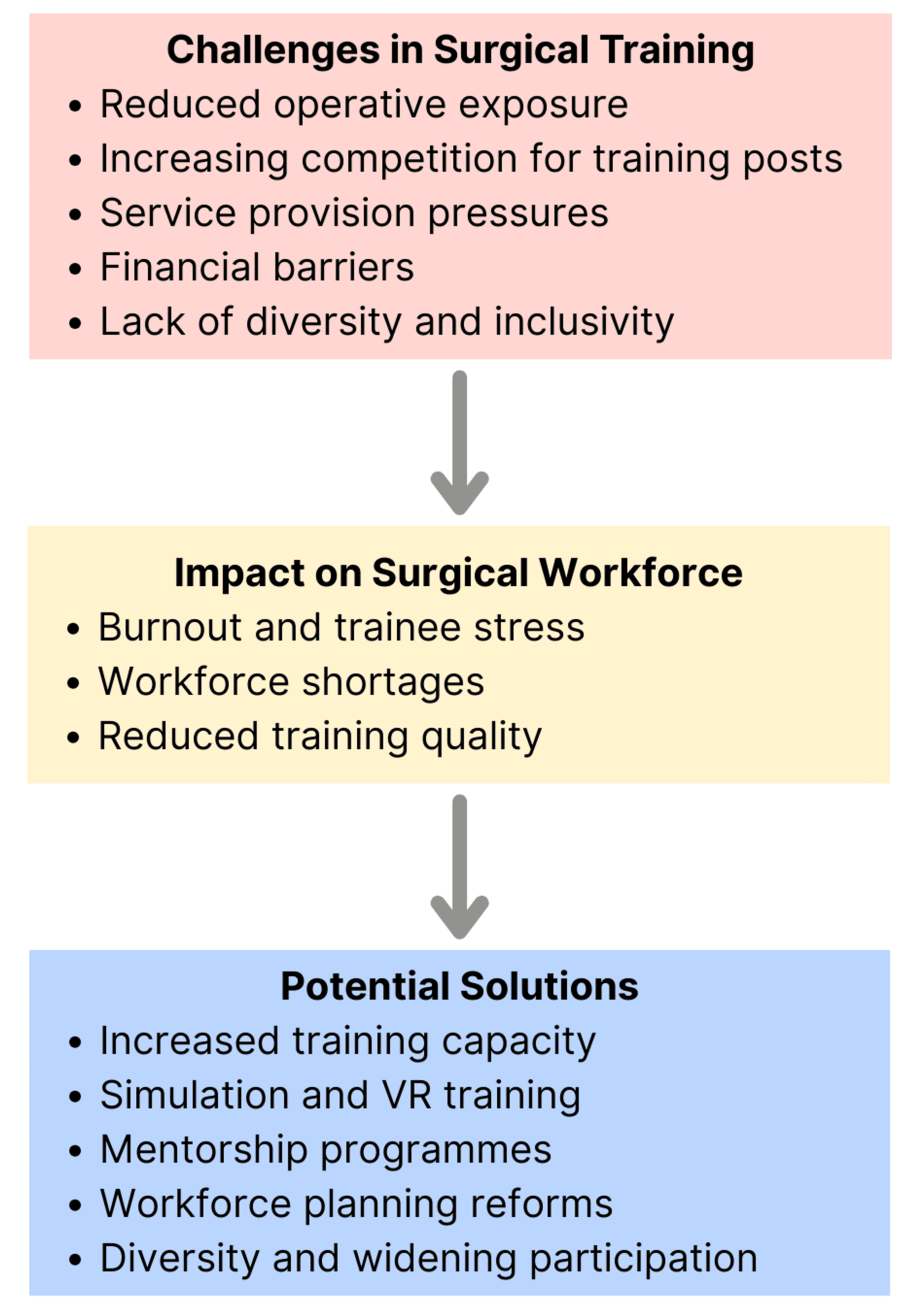
Conceptual framework illustrating challenges and potential solutions in UK surgical training. Conceptual framework of challenges and potential solutions in UK surgical training. This figure was independently created using information summarised from the cited sources [4,8,18,25,30]. The image was designed using Canva (Canva Pty Ltd., Sydney, Australia). VR: virtual reality.

The escalating cost for the NHS to train a surgeon has become a significant concern, with expenses reaching up to £230,000 from medical school through to surgical consultancy. As a result, trainees often contribute financially to support the continuation of their training. The personal financial cost to trainees can amount to approximately £40,000, making it challenging for individuals from disadvantaged socio-economic backgrounds to pursue a surgical career [29,30]. This financial disparity can further exacerbate existing socio-economic inequalities within the field. The key structural challenges affecting surgical training in the United Kingdom and potential approaches to address them are summarised in Figure 2.

Strategies to improve surgical training

Addressing these challenges requires a multifaceted approach involving educational reform, workforce planning, technological innovation, and stronger support systems for trainees. A comprehensive strategy should encompass improving support systems for surgical candidates, promoting diversity and inclusivity within the surgical workforce, and balancing supply and demand for training opportunities. By proactively addressing these issues, the surgical community can work towards building a resilient and diverse workforce that can meet the healthcare demands of the future effectively. Table 2 summarises these potential strategies.

**Table 2:** Proposed strategies to improve surgical training. Summary of proposed strategies to address challenges in surgical training in the UK. This table was independently created using information summarised from the cited sources [4,8,25,30].

Strategy	Description	Potential Benefit
Simulation-based training	Use of virtual reality and surgical simulators	Allows trainees to practice skills safely
Expansion of training posts	Increase available surgical training positions	Reduces competition bottlenecks
Mentorship programmes	Guidance from experienced surgeons	Improves trainee development and well-being
Flexible training pathways	Adapt training schedules to individual needs	Supports work-life balance
Workforce planning	Align training posts with future healthcare needs	Ensures a sustainable surgical workforce

Resolving challenges with traditional training

Examining the challenges posed by high service demands and time constraints faced by doctors is a crucial issue the NHS needs to overcome. The implementation of structured training programmes plays a pivotal role in overcoming these challenges by establishing clear pathways and realistic expectations of trainees' responsibilities [8]. However, the pressured nature of NHS service provision, coupled with unrealistic expectations for trainees, hinders the effectiveness of these programmes. The lack of dedicated study time further exacerbates the situation, making it challenging for trainees to meet these expectations [31]. Allocating additional study time, both within and outside regular working hours, could positively impact trainees, enabling them to consolidate their knowledge in a clinical setting. This would help balance service provision and training, allowing for a more effective and well-rounded learning experience.

This could be enhanced by seamlessly integrating training into the delivery of healthcare services. This integration could involve incorporating dedicated training sessions within clinical shifts, providing trainees with the opportunity to learn and contribute to patient care simultaneously. Such a holistic approach aims to optimise the training experience while addressing the practical demands of healthcare service provision.

Healthcare is on the brink of significant evolution, driven by pivotal trends in technology, educational methodologies, and the healthcare landscape. Technological integration is poised to be at the forefront, with virtual reality (VR) and artificial intelligence (AI) expected to transform medical training [32]. The rising prominence of remote training enables trainees to access educational resources and engage in collaborative learning experiences nationally. These advancements need to be adopted by the NHS for surgical trainees. The integration of advanced technologies into surgical training programmes addresses the need for a more immersive learning experience, allowing trainees to refine their skills in a safe and controlled environment whilst simultaneously gaining operating experience [33].

Providing flexibility in training pathways acknowledges the diverse needs and career goals of individual trainees. Recognising that not all trainees follow the same path or timeline allows for a personalised approach to training, accommodating different learning styles and aspirations within the surgical field. This flexibility supports trainees to have a healthy work-life balance [34].

Encouraging interdisciplinary training programmes within the NHS fosters collaboration among different healthcare professionals [35]. Exposure to multidisciplinary teams allows surgical trainees to gain valuable insights into holistic patient care and improve communication skills with colleagues from various specialities [36]. Integrating non-technical skills, such as communication and teamwork, into surgical training further supports holistic skill development.

Implementing a system for the regular review and adaptation of surgical training programmes is crucial to ensure they remain aligned with evolving healthcare needs and advancements [37]. This iterative approach allows the NHS to continually enhance the quality and relevance of surgical education.

Through recognising the importance of trainee well-being, the NHS can implement targeted support programmes, including initiatives focused on mental health, stress management, and work-life balance. Creating a supportive environment is crucial for the overall success and satisfaction of surgical trainees and could help address challenges such as high dropout rates and burnout [38].

In summary, the future of surgical training is dynamic, embracing technological innovations, personalised approaches, and a commitment to continuous improvement. This evolution aims to produce highly skilled, adaptable surgeons capable of meeting the evolving challenges of healthcare.

Challenging competition ratios

There are several strategies which may be considered to overcome the challenges. Firstly, to address the critical issue of limited surgical training positions, the NHS must strategically focus on increasing training capacity. Expanding the number of available positions, provided there are sufficient resources and infrastructure, can alleviate the limited availability of training posts and reduce competition among candidates. The shortage of surgical training posts poses a significant obstacle for potential applicants. Effectively managing the balance between the supply and demand for surgical training positions requires strategies that can mitigate excessive competition and stress among candidates. This approach requires not only the enhancement of existing training models but also engagement with healthcare policy decision-makers to drive systemic changes and ensure a sustainable future for surgical education.

Expanding training capacity and improving existing training models may help alleviate the lack of training opportunities while also creating a more adaptive system that can meet the changing demands of the healthcare sector. To drive systemic changes that will safeguard the future of surgical education, it is essential to engage with decision-makers in healthcare policy. This engagement should underscore the potential consequences of maintaining the status quo. Furthermore, while increasing NHS funding from the private sector could potentially secure more training positions, such implementations require several years to materialise [39].

Secondly, resources such as clinical sites and mentorship should be effectively allocated to maximise the capacity of existing training programmes. A potential solution to the rising cost of surgical training has emerged following the coronavirus pandemic. Affordable and accessible surgical solutions in the form of tools such as portable simulators or modulators have been trialled. These innovations aim to offset the significant costs associated with traditional methods and enable trainees to practise at home. Additionally, it is crucial to explore cost-effective alternatives to training methods utilised in lower-income countries, as they can be applied to the UK, contributing to cost reduction while maintaining high training standards [40,41]. For example, the GlobalSurgBox is a modular training device housed in a toolbox, costing less than $10 per unit. This device is designed to address individual learning needs and reinforce fundamental skills encountered in surgical training [30]. A study involving trainees from the US, Kenya, and Rwanda demonstrated that limited access to simulation was a barrier to practise and that GlobalSurgBox helped reduce these barriers. Participants found it convenient and stated that it better prepared them for clinical settings [42,43]. Additionally, a quality improvement programme in Scotland loaned portable laparoscopic simulators to three consecutive years of new core surgical trainees. Most trainees stated that they would recommend the programme to others; however, perceived support from local trainers was lacking. This underlines the importance of not only implementing innovative training solutions but also ensuring adequate support structures are in place to maximise programme effectiveness [44].

Implementing robust support systems and mentorship programmes that allow trainees to work with experienced surgeons, even if they can't secure a formal training position immediately, is vital [45]. These programmes provide guidance, support, and feedback, enhancing professional development, promoting inclusivity [18,46] and increasing trainee satisfaction and retention rates [30]. They can also contribute to a more encouraging and nurturing learning environment, reducing burnout and supporting the well-being of future surgeons [46].

The development of shorter, more focused training programmes could further improve training by imparting specific surgical skills or competencies [47]. These innovative models aim to provide targeted training in emerging techniques or technologies, ensuring that surgical trainees remain at the forefront of advancements in their field [48]. This approach fosters a more flexible and dynamic training experience, adapting to the evolving needs of surgical practice.

The consideration of regional training centres is another strategic initiative. The establishment of these centres seeks to decentralise training opportunities, making high-quality education more accessible to trainees across various regions [49]. By creating centres outside large urban areas, this approach addresses geographical disparities in training, promoting a more widespread distribution of surgical expertise and enhancing healthcare delivery in diverse settings.

Conducting comprehensive workforce planning is paramount in addressing the challenge of limited surgical training positions. This process entails a systematic analysis of current and future demands for surgical services, considering factors such as population growth, demographic shifts, and advancements in medical technology. Aligning the number of training positions with anticipated healthcare workforce needs ensures a balanced and adequately skilled surgical workforce.

Addressing the challenges associated with limited surgical training positions requires a holistic and proactive approach. This requires continuous evaluation of the healthcare landscape, flexibility in training structures, and a commitment to innovation and adaptability.

Challenging equality, diversity, and inclusion

By providing access to mentors who have navigated similar experiences, these programmes inspire and encourage aspiring surgeons, especially those from underrepresented backgrounds, thereby fostering greater diversity within the field [30]. However, the competition within surgical selection tends to favour candidates from higher socio-economic backgrounds, perpetuating the association of surgical positions with social privilege [50]. Diversifying role models from various backgrounds can encourage positive change [51].

To further address the lack of diversity within the surgical workforce, widening participation is key. This begins with medical schools, providing accessible resources and experiences to students from underprivileged backgrounds to strengthen their medical school applications. The commitment to diversity should extend throughout medical school with the aim of supporting speciality training applications. Additional efforts to bridge the networking gap for first-in-family medical students and create mentorship networks can encourage diversity within the field [26]. The RCS England published the Kennedy Report to help encourage diversity and made a 16-point plan as guidance. This included a mentorship scheme, collecting data to highlight areas that need action to increase fair inclusivity, providing seed funding and support to organisations that seek to address diversity and inclusion in surgery [52]. Furthermore, the RCS also started a Medical School Engagement Pilot Programme, which aims to engage medical students and promote surgery as a realistic and attractive career for students from diverse backgrounds [53]. A further initiative to widen diversity in surgery is the Women in Surgery network, which aims to support and inspire women in surgery [21].

Future directions in surgical training

Modern training programmes, such as proficiency-based progression [54,55], have demonstrated their ability to enhance patient safety and reduce errors in various surgical procedures, addressing the need for a 21st-century evolution of the traditional apprenticeship model [54,55]. These newer approaches prioritise achieving specific skills and milestones over the traditional emphasis on the duration of training [56]. Trainees are guided to progress by demonstrating proficiency at well-defined milestones and are required to demonstrate proficiency before advancing to more complex procedures. This method not only ensures a more standardised and efficient training process but also contributes to reducing errors and enhancing patient safety [54].

Moreover, technological innovations have revolutionised surgical training. VR and simulation-based training have emerged as valuable tools for trainees to overcome the challenges of limited access to the operating room. VR allows aspiring surgeons to immerse themselves in realistic surgical scenarios, offering a safe environment to practise and refine their skills [4]. Similarly, simulation-based training provides hands-on experience with surgical instruments and techniques using advanced simulators, helping trainees gain confidence and competence in a risk-free setting [55]. Multiple studies have shown how this technology can enhance surgical abilities and shorten the learning curve for trainees [25,57]. These educational advancements and technological breakthroughs collectively contribute to a more comprehensive and dynamic approach to surgical training, complementing the traditional apprenticeship model [27].

As a result of these various factors, the apprenticeship style of surgical education must adapt and evolve to meet the changing demands of the healthcare landscape. Integrating elements of newer educational techniques and leveraging technology may prove crucial in maintaining the effectiveness of the apprenticeship model while addressing the challenges posed by sub-specialisation, increased competition, and extended training durations. By striking a balance between tradition and innovation, surgical training can continue to produce skilled and competent surgeons capable of meeting the diverse and evolving needs of patients and healthcare systems.

## Conclusions

Surgical training in the UK faces significant challenges, including reduced operative exposure, rising competition for training posts, workforce pressures, those without money losing coaching opportunities for good courses, and persistent issues in diversity and inclusion. While the traditional apprenticeship model remains a key foundation of surgical education, it must continue to evolve in response to modern healthcare demands. Expanding training capacity, strengthening mentorship and support systems, improving workforce planning, and incorporating simulation and other technological innovations can help address current limitations in surgical training. By combining traditional experiential learning with modern educational strategies, the UK surgical training system can continue to develop a skilled, resilient, and sustainable surgical workforce capable of meeting the evolving needs of healthcare.
